# Quality of Life among University Students with Premenstrual Symptoms: The Role of Emotion Regulation

**DOI:** 10.1192/j.eurpsy.2023.1349

**Published:** 2023-07-19

**Authors:** A. Ben Elazar, L. Canetti, M. Azoulay, R. Dan, G. Goelman, R. Segman, C. Kalla, O. Bonne, I. Reuveni

**Affiliations:** 1 Faculty of Medicine, The Hebrew University of Jerusalem; 2 Department of Psychiatry, Hadassah Hebrew University Medical Center; 3 Department of Psychology, The Hebrew University of Jerusalem; 4 Department of Neurology, Hadassah Hebrew University Medical Center; 5 Edmond and Lily Safra Center for Brain Sciences (ELSC), The Hebrew University of Jerusalem, Jerusalem, Israel

## Abstract

**Introduction:**

Premenstrual dysphoric disorder (PMDD), a severe form of the premenstrual syndrome (PMS), negatively impacts women’s quality of life, including physical and mental aspects. Difficulties in emotion regulation, more prevalent among women with PMDD, are also associated with poor quality of life.

**Objectives:**

To determine whether the negative impact of premenstrual symptoms on quality of life is partially explained by emotional dysregulation.

**Methods:**

A total of 112 women completed self-report questionnaires, including a demographic questionnaire, the Premenstrual Symptoms Screening Tool (PSST), Medical Outcomes Study Short Form-36 (SF-36), and the Difficulties in Emotion Regulation Scale (DERS). To test the mediation hypothesis, direct and indirect effects of premenstrual symptoms on quality of life were calculated.

**Results:**

Quality of life was impaired in the PMS/PMDD group compared to controls. The PMS/PMDD group showed significantly greater emotion regulation difficulties as compared to the No/mild PMS group. Emotion regulation difficulties partially mediates the relationship between premenstrual symptoms and quality of life, for both SF-36 total score and mental subscale, but not for physical subscale.

**Image:**

**Figure:**
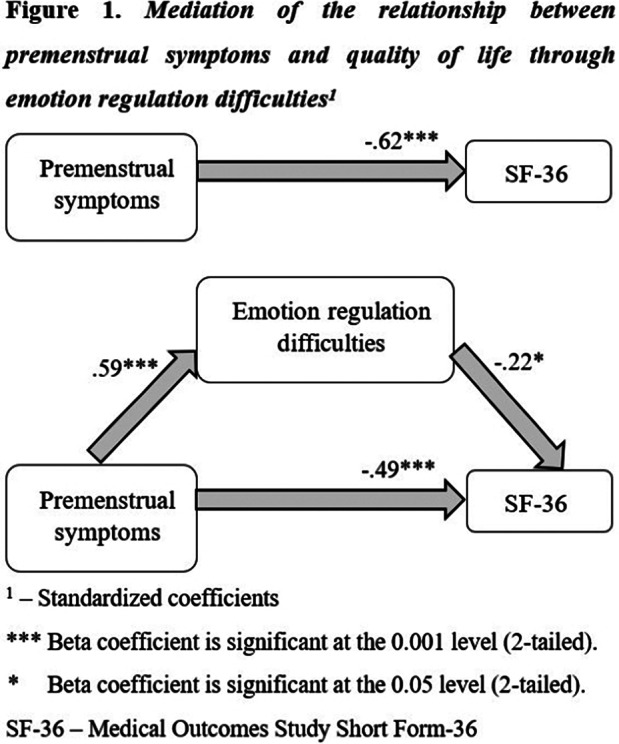

**Image 2:**

**Figure:**
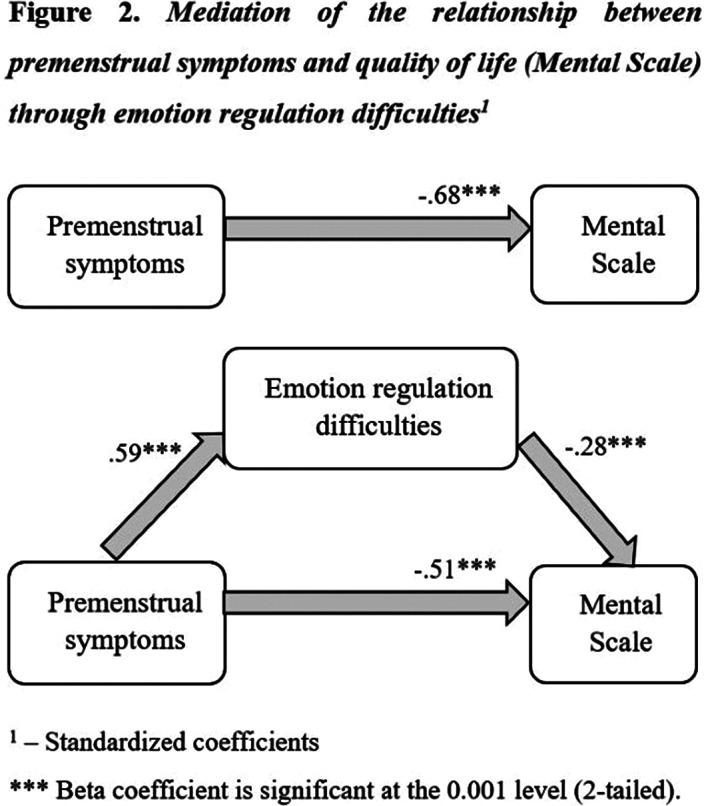

**Conclusions:**

Emotion regulation difficulties could be a possible target for interventions that could improve the quality of life among women who experience premenstrual symptoms.

**Disclosure of Interest:**

None Declared

